# Boron-incorporating hemagglutinating virus of Japan envelope (HVJ-E) nanomaterial in boron neutron capture therapy

**DOI:** 10.1080/14686996.2019.1586051

**Published:** 2019-03-29

**Authors:** Shuichiro Yoneoka, Yasuhiro Nakagawa, Koichiro Uto, Kazuma Sakura, Takehiko Tsukahara, Mitsuhiro Ebara

**Affiliations:** a Laboratory for Advanced Nuclear Energy, Tokyo Institute of Technology, Tokyo, Japan; b International Center for Materials Nanoarchitectonics (WPI-MANA), National Institute for Materials Science (NIMS), Tsukuba, Ibaraki, Japan; c Graduate School of Pure and Applied Science, University of Tsukuba, Tsukuba, Ibaraki, Japan; d Graduate School of Engineering, The University of Tokyo, Tokyo, Japan; e Innovation Center of NanoMedicine, Kawasaki Institute of Industrial Promotion, Kawasaki-ku, Kawasaki, Japan; f Department of Medical Innovation, and Respiratory Center, Osaka University Hospital, Suita, Osaka, Japan; g Graduate School of Industrial Science and Technology, Tokyo University of Science, Katsushika-ku, Tokyo, Japan

**Keywords:** Hemagglutinating virus of Japan-envelope (HVJ-E), boron neutron capture therapy (BNCT), benzoxaborole, surface modification, hemolysis, 20 Organic and soft materials (colloids, liquid crystals, gel, polymers), 101 Self-assembly / Self-organized materials, 212 Surface and interfaces, 501 Chemical analyses, polymer, bio nanomaterial

## Abstract

Combining immunotherapeutic and radiotherapeutic technique has recently attracted much attention for advancing cancer treatment. If boron-incorporated hemagglutinating virus of Japan-envelope (HVJ-E) having high membrane fusion ability can be used as a boron delivery agent in boron neutron capture therapy (BNCT), a radical synergistic improvement of boron accumulation efficiency into tumor cells and antitumor immunity may be induced. In this study, we aimed to develop novel boron-containing biocompatible polymers modified onto HVJ-E surfaces. The copolymer consisting of 2-methacryloyloxyethyl phosphorylcholine (MPC) and methacrylamide benzoxaborole (MAAmBO), poly[MPC-*co*-MAAmBO], was successfully synthesized by using a simple free radical polymerization. The molecular structures and molecular weight of the poly[MPC-*co*-MAAmBO] copolymer were characterized by nuclear magnetic resonance and matrix-assisted laser desorption ionization time-of-flight mass spectrometry, respectively. The poly[MPC-*co*-MAAmBO] was coated onto the HVJ-E surface via the chemical bonding between the MAAmBO moiety and the sugar moiety of HVJ-E. DLS, AFM, UV-Vis, and fluorescence measurements clarified that the size of the poly[MPC-*co*-MAAmBO]-coated HVJ-E, HVJ-E/p[MPC-MAAmBO], to be about 130 ~ 150 nm in diameter, and that the polymer having 9.82 × 10^6 ~ 7^ boron atoms was steadily coated on a single HVJ-E particle. Moreover, cellular uptake of poly[MPC-*co*-MAAmBO] could be demonstrated without cytotoxicity, and the hemolysis could be successfully suppressed by 20%. These results indicate that the HVJ-E/p[MPC-MAAmBO] may be used as boron nanocarriers in a combination of immunotherapy with BNCT.

## Graphical Abstract

**Figure:**
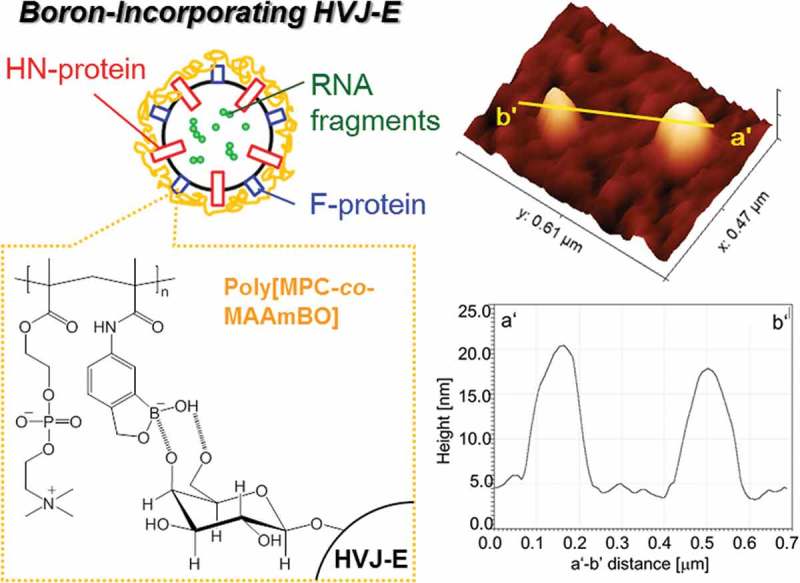

## 1. Introduction

Cancer immunotherapy has been approved as an advanced therapeutic technique, and the effectiveness of several immune checkpoint inhibitors for various types of cancer such as melanoma, nonsmall cell lung cancer, etc. has been proven [1–3]. Recently, a new immunotherapeutic strategy using inactivated Hemagglutinating Virus of Japan Envelope (HVJ-E) as a gene delivery vector has attracted much attention, because HVJ-E has high membrane fusion ability induced by fusion (F) and hemagglutinin-neuraminidase (HN) proteins on the surface [3–5]. Previous studies have found that the membrane fusion ability of HVJ-E activates the antitumor immunity by inducing interleukin (IL)-6 and C-X-C motif chemokine (CXCL) 10 expression in dendritic cells, and more than half of the tumors in mice can thus be destroyed [6,7]. Induction of tumor-selective apoptosis is also produced by the membrane fusion, since the viral RNA genome fragments of HVJ-E activate the retinoic acid-inducible gene-I (RIG-I) signaling pathway and upregulate the tumor necrosis factor-related apoptosis-inducing ligand (TRAIL) and Noxa [8]. Moreover, HVJ-E incorporating antitumor immunotherapeutic agents have been demonstrated to be useful as a drug-delivery carrier for cancer treatment [9–12]. However, there is a serious problem in that membrane fusion of HN proteins with erythrocyte on the HVJ-E surface often occurs, resulting in hemagglutination and hemolysis [13]. In order to overcome these disadvantages, surface coating of HJV-E which can minimize the hemagglutination and the nonspecific adsorption has been investigated by some groups including ourselves, and HVJ-E surfaces have been successfully coated with polymeric materials by means of cationized-gelatin conjugation and layer-by-layer (LbL) assembly methods [14–16].

Radiation stimulates cytotoxic T lymphocyte (CTL) activity, and leads to not only systemic antitumor immune response but also growth suppression of nonirradiated metastatic tumors at distant sites from irradiated primary tumor sites. This phenomenon called the abscopal effect has been known to be facilitated by radiotherapy in combination with immunotherapy [17–19]. Recent studies have revealed that the combination of immunotherapy with radiotherapy enables the synergistic enhancement of cancer-treatment efficacy, and global clinical trials for some types of cancer are ongoing [20]. Among the radiation therapy, boron neutron capture therapy (BNCT) is a powerful cancer cell-targeted radiation treatment. The nuclear reaction of thermal neutrons and boron-10 isotopes (^10^B, 19.9% natural abundance) emits alpha (α) particles and lithium atoms because of the ^10^B(n,α)^7^Li reaction. Since the range distance of the particles, ca. 10 µm, is quite close to the size of single cell, the selective destruction of target tumor cells can be accomplished without any effect on normal cells [21]. Low molecular weight boron agents such as sodium borocaptate (BSH) and p-boronophenylalanine (BPA) have been clinically used as boron-containing pharmaceuticals [22,23], while they exhibit a short-term retention time in tumor cells, and often cause vascular endothelial injuries because of the severe increase of boron concentrations in blood [23–29]. Various boron-incorporated macromolecular agents with high boron contents such as liposomes, polymeric nanomicelles, and antibodies have also been produced, and their physiological-pathophysiological evaluation has been performed [30–36]. However, the macromolecular approaches are not satisfactory in terms of the insufficient boron accumulation efficiency in tumor cells, low boron content per unit weight, and the complicated synthesis.

For overcoming these disadvantages, Fujii et al. has applied HVJ-E to boron delivery carriers for BNCT treatment by encapsulating BSH into the cationized gelatin conjugate HVJ-E (CG-HVJ-E-BSH). The results have shown that CG-HVJ-E-BSH has both high tumor/normal liver cells ^10^B concentration ratio and high retention in the tumor cells, and can radically enhance the therapeutic efficacy without normal liver injury as compared with BSH itself [15]. Accordingly, utilization of boron-incorporating HVJ-E vector is expected to open the door of pharmaceutical breakthrough in BNCT treatment, since the vector induces the synergistic effect of combining drug delivery and immune activity. In spite of its great potential, there has been no other research on the boron-incorporating HVJ-E. The development of novel boron-labelled HVJ-E vector is also quite challenging.

If the boron-labelled HVJ-E can be used as a potential radioimmunotherapeutic agent for BNCT, a radical synergistic improvement of boron accumulation efficiency into tumor cells and enhancement of the abscopal effect may be induced. Moreover, it is expected that modification of boron-containing polymers on HVJ-E surfaces can not only achieve high loading of boron atoms and hemolysis suppression but also provide anti-cancer therapeutic functions by encapsulating anticancer agents inside the surface-modified HVJ-E. Therefore, in this study, we aimed to synthesize by means of simple polymerization and surface modification techniques a biocompatible boron-containing copolymer-coated HVJ-E which allows to be a functional nanocarrier for BNCT.

**Figure 1.**
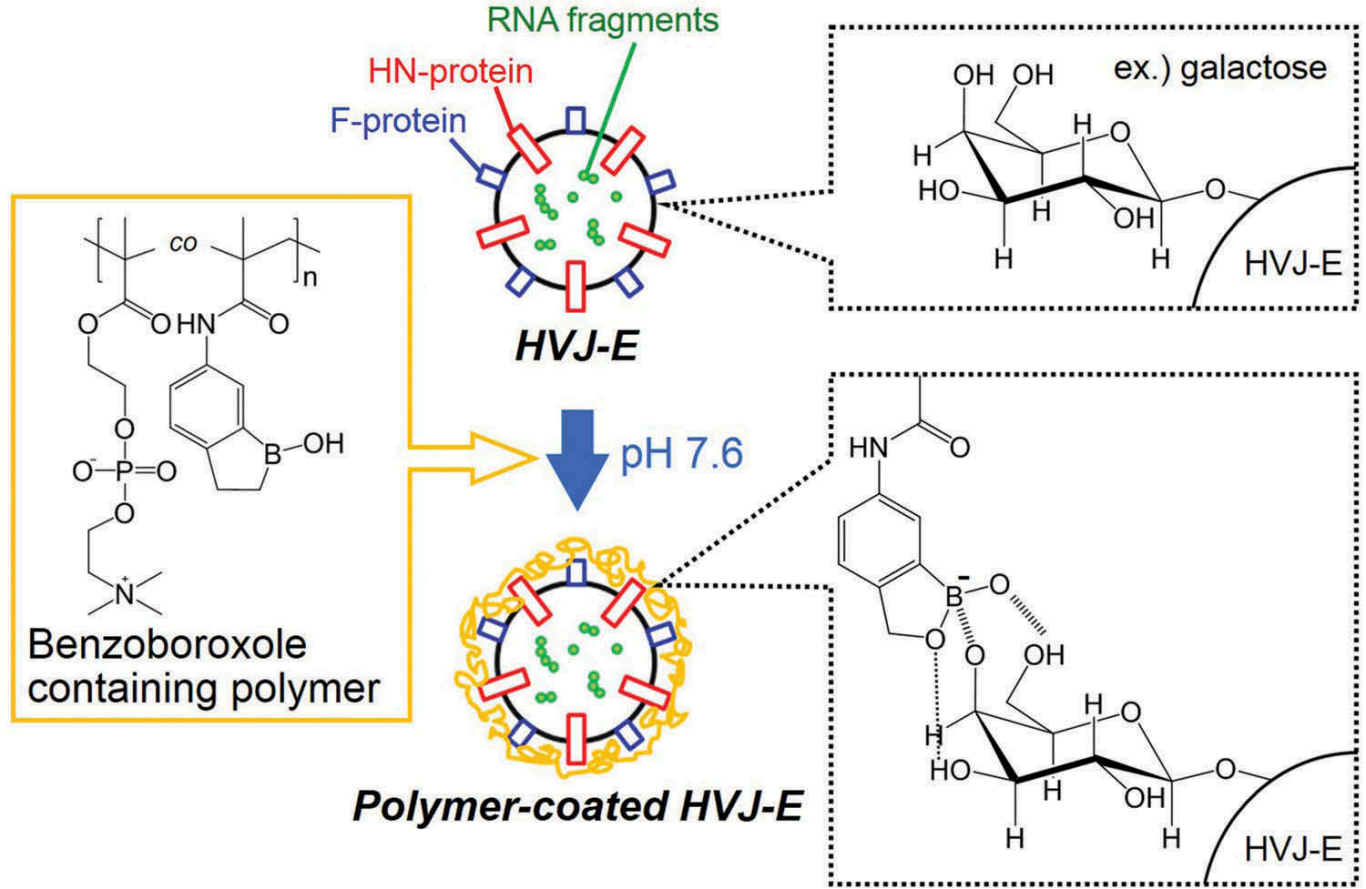
Schematic illustration of HVJ-E surface modification by benzoxaborole-containing polymer.

The boronated copolymer is composed of poly(2-methacryloyloxyethyl phosphorylcholine) (MPC) with methacrylamide benzoxaborole (MAAmBO), termed as poly[MPC-*co*-MAAmBO]. As shown in our conceptual illustration (Figure 1), since benzoxaborole (BO) is capable of binding to monosaccharides such as galactose in neutral buffer solution with pH above 7.6 because of the formation of ionized B(OH)_2_^–^ groups of BO, the poly[MPC-*co*-MAAmBO] should be modified onto HVJ-E surfaces [37–42]. The synthesized poly[MPC-*co*-MAAmBO] was characterized by ^1^H and ^11^B nuclear magnetic resonance (NMR), matrix-assisted laser desorption ionization time-of-flight mass spectrometry (MALDI-TOF MS), dynamic light scattering (DLS), and atomic force microscope (AFM) measurements.

The poly[MPC-*co*-MAAmBO] was modified onto HVJ-E surfaces, and the amount of boron atoms and the stability of the polymer coated on HVJ-E were evaluated by using UV-Vis and fluorescence spectroscopic analyses. Moreover, the effects of the poly[MPC-*co*-MAAmBO] on hemolysis, cellular viability, and cellular uptake were also examined.

## 2. Experiment

### 2.1. Materials

MPC and fetal bovine serum (FBS) were purchased from Sigma-Aldrich (St Louis, MO, USA), and used as received. Methanol-d_4_ (CD_3_OD; 99.96 at.%D), deuterium oxide (D_2_O; 99.96 at.%D), and dimethyl sulfoxide-d_6_ (DMSO-d_6_; 99.9 at.%D) were obtained from Acros Organics (Morris Plains, NJ, USA). 3-(trimethylsilyl)-1-propanesulfonic acid-d_6_ sodium salt (DSS-d_6_) and D(+)-glucose were obtained from Wako Pure Chemical Co. Ltd. (Osaka, Japan). 5-amino-2-(hydroxymethyl)phenylboronic acid cyclic monoester, methacryloyl chloride, and 4,4ʹ-azobis(4-cyanovaleric acid) (ACVA) were obtained from Tokyo Chemical Industry Co. Ltd. (Tokyo, Japan). Cyanine5 amine dye was obtained from Lumiprobe Corporation (Hunt Valley, MD, USA). Other chemical reagents were purchased from Kanto Chemical (Tokyo, Japan) and used as received. Freeze-dried HVJ-E Vector Kit of GenomeONE™-NEO series and Chicken blood were obtained from Ishihara Sangyo Kaisha Ltd. (Osaka, Japan) and Nippon Bio-Test Laboratories Inc. (Tokyo, Japan), respectively. Dulbecco’s Phosphate Buffered Saline without Ca and Mg (D-PBS(-)) was purchased from Nacalai Tesque, Inc. (Kyoto, Japan). FluoroBrite Dulbecco’s modified eagle medium (DMEM) and antibiotic-antimycotic (Anti-Anti) (100X) were purchased from Gibco (Grand Island, NY, USA).

### 2.2. Preparation of poly[MPC] and poly[MPC-co-MAAmBO]

MAAmBO (216.8 g mol^–1^) was synthesized as follows. 1.7 ~ 1.8 M NaOH aqueous solution was cooled down to below 1.0 °C, and 1.0 g of 5-amino-2-(hydroxymethyl)phenylboronic acid cyclic monoester (6.7 mmol) was added and dissolved. After that, 0.98 mL of methacryloyl chloride (13.5 mmol) was very gently dropped into the solution during vigorous stirring, and the reaction was proceeded for 2 h under light shielding conditions. After a concentrated HCl solution was added slowly until pH equaled 2 ~ 3, pale-brown-colored MAAmBO precipitates could be recovered. The precipitates were filtrated and dried in vacuum overnight at 30 °C.

Poly[MPC] and poly[MPC-*co*-MAAmBO] were synthesized by using free radical polymerization method. MPC (295.3 g mol^–1^) and MAAmBO as monomers and ACVA as a radical initiator were simultaneously added in a polypropylene tube. The amount of the added monomers and initiator is listed in Table 1. After 10 mL of ethanol degassed in an Ar gas bubbling was added in the reaction tubes, the monomers and initiator were dissolved. The solutions were heated up to 69 °C and reacted for 24 h under Ar-filled glove box. After the polymerization, the resulting solutions were reduced in volume using a rotary evaporator, and dialyzed using regenerated cellulose membrane filters (Spectra/Pro 6, MWCO: 1 kDa, Spectrum Laboratories, Inc.) in methanol and distilled water. The dialyzed solution was lyophilized, and fine powder of poly[MPC] (white) or poly[MPC-*co*-MAAmBO] (pale yellow) was successfully recovered.

**Table 1.** Amount of MPC, MAAmBO, and ACVA in feed solutions in polymerization of poly[MPC] and poly[MPC-*co*-MAAmBO], respectively.

Polymer	In feed weights		
	MPC	MAAmBO	ACVA
Poly(MPC)	2.95 g (9.99 mmol)	–	11.2 mg (40.0 μmol)
Poly(MPC-co-MAAmBO)	2.09 g (7.07 mmol)	651 mg (3.00 mmol)	11.2 mg (40.0 μmol)

### 2.3 Characterization of poly[MPC] and poly[MPC-co-MAAmBO]

The molecular structures of the synthesized MAAmBO, poly[MPC], and poly[MPC-*co*-MAAmBO] were examined by using ^1^H and ^11^B-NMR spectroscopy (JEOL JNM-ECX400P; 400 MH and 125 MHz for ^1^H and ^11^B, respectively) at ambient temperature. For the determination of ^1^H- and ^11^B-NMR chemical shifts, DSS-d_6_ and boron trifluoride diethyl ether complex (BF_3_·Et_2_O) were adapted as an internal standard and an external standard, respectively. The deuterated solvents including D_2_O, CD_3_OD, and DMSO-d_6_ were used for shimming and locking. The MALDI-TOF MS measurement (MALDI-TOF MS; ultrafleXtreme, Bruker Corp.) was used to determine roughly the number-average molecular weight (*M*_n_) of the synthesized polymers. 2ʹ,4ʹ,6ʹ-Trihydroxy acetophenone (THAP) was utilized as a matrix.

### 2.4. Preparation of polymer-modified HVJ-E

Poly[MPC] or poly[MPC-*co*-MAAmBO] of various concentrations (1, 10, 25, 50, 100, 200 mg mL^−1^) was individually prepared in 9.5 mM PBS (pH7.6). 4 packs (ca. 15 mg) of freeze-dried HVJ-E were suspended in 800 μL of 9.5 mM PBS (pH 7.6), and each suspension was stored in a cool dry place. 5 μL of HVJ-E suspension was mixed with 45 μL of polymer solution in 1.5 mL tips, and the solution was kept in 4 °C for about 50 min to coat poly[MPC] or poly[MPC-*co*-MAAmBO] onto the HVJ-E surfaces. Both the polymer-coated HVJ-E (HVJ-E/p[MPC] and HVJ-E/p[MPC-MAAmBO]) were collected by a centrifuge (14,000 rpm) for 10 min at 4 °C, and the supernatant was removed. Uncoated polymers were removed by repeatedly centrifuging in 500 μL of 9.5 mM PBS (pH 7.6) suspension containing the polymer-coated HVJ-E. In order to confirm the polymer modified onto HVJ-E, cyanine5 (Cy5) amine dye-functionalized poly[MPC] and poly[MPC-*co*-MAAmBO] were prepared. 96 mg of poly[MPC] or poly[MPC-*co*-MAAmBO] were dissolved in 12 mL ethanol. 3.82 mg (5.84 μmol) of Cy5 amine in 1 mL ethanol and 3.83 mg (20.0 μmol) of 1-(3-dimethylaminopropyl)-3-ethylcarbodiimide hydrochloride (EDC HCl) in 1 mL ethanol were simultaneously added to the polymer solution. The solutions were stirred vigorously for 12.5 h under light shielding conditions. The Cy5 amine was bound with the terminal carboxyl groups of the polymers by dehydration and condensation reactions. After the modification, unreacted Cy5 amine was removed by dialysis in methanol and distilled water, and the recovered solution was lyophilized, resulting in a poly[MPC]-Cy5 or poly[MPC-*co*-MAAmBO]-Cy5 pale-blue fine powder.

### 2.5. Characterization of polymer-modified HVJ-E

10 μL of HVJ-E, HVJ-E/p[MPC], and HVJ-E/p[MPC-MAAmBO] was suspended in 1 mL of 9.5 mM PBS (pH 7.6), and each particle size was determined by DLS measurement (Delsa^TM^ Nano HC, Beckman Coulter Inc., USA) at 20 °C. Moreover, the suspension containing HVJ-E/p[MPC-MAAmBO] was dropped onto a hydrophobic surface, and the shapes and sizes of HVJ-E/p[MPC-MAAmBO] were measured by an AFM (NaioAFM, Nanosurf AG.).

The polymer modified HVJ-E was confirmed using the Cy5-modified polymers. Cy5-modified poly[MPC] or Cy5-modified poly[MPC-*co*-MAAmBO] (100 mg mL^−1^) were dissolved in 9.5 mM PBS (pH 7.6). 5 μL of HVJ-E suspension was mixed with the polymer solution (45 μL) in 1.5 mL tips, and was kept at 4 °C for about 50 min to coat the polymer onto an HVJ-E surface. The Cy5-modified polymer-coated HVJ-E (HVJ-E/Cy5-p[MPC] or HVJ-E/Cy5-p[MPC-MAAmBO]) was collected by centrifuge (14,000 rpm) for 10 min at 4 °C and the supernatant was removed. The purified HVJ-E/Cy5-p[MPC] or HVJ-E/Cy5-p[MPC-MAAmBO]) (100 μL) was suspended in 9.5 mM PBS (pH7.6), and the absorbance spectrum was observed by a microplate reader (Infinite® 200 PRO, Tecan Japan Co., Ltd., Japan) equipped with a 96-well microplate (IWAKI, Tokyo, Japan).

The amount of boron atoms in the polymer modified on each HVJ-E was calculated from quantitative fluorescence analysis of HVJ-E/Cy5-p[MPC-MAAmBO]. 135 μL of D-PBS(-) solution containing Cy5-poly[MPC-*co*-MAAmBO] (50 mg mL^–1^) was mixed with 15 μL of HVJ-E suspension, which includes approximately 4.50 × 10^8^ ~ 10^9^ particles of HVJ-E, at 4 °C for 50 min. The resulting solution was repeatedly washed with D-PBS(-), and the polymer-coated HVJ-E (HVJ-E/Cy5-p[MPC-MAAmBO]) was recovered by centrifugation at 4 °C. The recovered HVJ-E/Cy5-p[MPC-MAAmBO] was suspended in 2.35 mL of D-PBS(-), and the fluorescence emission spectrum was measured at the excitation wavelength of 642 nm by FP-8500 (JASCO, Tokyo, Japan). From the fluorescence intensity obtained at 657 nm and the calibration curve, the amount of boron atoms in the polymer was evaluated.

In order to clarify surface-coating stability of poly[MPC-*co*-MAAmBO] on HVJ-E, effects of glucose on HVJ-E/Cy5-p[MPC-MAAmBO] were examined by immersing in 2 mg mL^–1^ glucose solution which is higher than normal blood glucose level (0.7 ~ 1 mg mL^–1^). 10 μL of D-PBS(-) solution containing HVJ-E/Cy5-p[MPC-MAAmBO] (50 mg mL^–1^) was suspended in 500 μL of D-PBS(-) with 2 mg mL^−1^ D-glucose or without D-glucose, and each solution was kept at 4 °C for about 30 minutes. The HVJ-E/Cy5-p[MPC-MAAmBO] was collected by centrifuge (14,000 rpm) for 10 min at 4 °C, and the supernatant was removed. After the process was repeated three times, the recovered HVJ-E/Cy5-p[MPC-MAAmBO] was suspended in 100 μL D-PBS(-). UV-Vis absorbance spectrum of the solution containing the glucose-treated HVJ-E/Cy5-p[MPC-MAAmBO] was measured by a microplate reader equipped with 96-well microplate (*N* = 5).

### 2.6. Hemolysis test

About 5 mL of whole chicken blood was dispersed in 40 mL D-PBS(-), and centrifuged at 1000 rpm for 20 min and at 4 °C. After the supernatant was removed, only the precipitate was suspended again in 40 mL D-PBS(-) for washing. By repeating this procedure three to five times, the chicken erythrocyte was thoroughly purified. 8% chicken erythrocyte suspension was prepared by mixing 4 mL of the purified erythrocyte precipitate in 46 mL D-PBS(-). The suspension was stored in a refrigerator. Each HVJ-E, HVJ-E/p[MPC], or HVJ-E/p[MPC-MAAmBO] sample (10 μL) with polymer concentrations of 1, 10, 25, 50, 100, and 200 mg mL^–1^ was mixed with 8 vol% chicken erythrocyte suspension (500 μL), and the samples were incubated at 37 °C for 3 h. After incubation, each suspension was centrifuged at 2000 rpm for 5 min and at 4 °C and the supernatant was recovered, and the absorbance of the recovered supernatant was measured at 542 nm for the evaluation of hemolysis (*N* = 3).

### 2.7. Evaluation of cell viability

HepG2 cells were seeded on a 96 well microplate (1.0 × 10^4^ cells/well) in 200 μL FluoroBrite DMEM supplemented with 10% FBS and 1% Anti-Anti at 37 °C under 5% CO_2_. After overnight incubation, the medium was exchanged to DMEM/D-PBS(-) (= 9:1) containing 1 mg mL^−1^ poly[MPC-*co*-MAAmBO], and the cells were incubated in the same condition for 24 h. Alamar blue assay was adopted to evaluate cellular viability. 20 μL of alamarBlue® reagent (Invitrogen, Carlsbad, CA, USA) was added in each well, and the cells were incubated again in the same condition for 2 h. The fluorescence intensity at 585 nm exited by 540 nm (*N* = 5) of the HepG2 cells treated with poly[MPC-*co*-MAAmBO] was measured, and compared with that of control HepG2 cells without poly[MPC-*co*-MAAmBO].

### 2.8. Evaluation of cellar uptake of poly[MPC-co-MAAmBO]

HepG2 cells were seeded on a 24 well microplate (IWAKI, Tokyo, Japan) (5.0 × 10^4^ cells/well) in 500 μL FluoroBrite DMEM supplemented with 10% FBS and 1% Anti-Anti at 37 °C under 5% CO_2_. After overnight incubation, the medium was exchanged to DMEM/D-PBS(-) (= 49:1) containing 1 mg mL^–1^ fluorescence-labelled Cy5-poly[MPC-*co*-MAAmBO]. The cells were incubated in the same condition for 45 or 90 min, and rinsed three times by D-PBS(-). After incubation of HepG2 cells with the Cy5-poly[MPC-*co*-MAAmBO], the cellular uptake was observed by using fluorescence microscopy.

## 3. Results and discussion

### 3.1. Characterization of MAAmBO, poly[MPC], and poly[MPC-co-MAAmBO]

**Figure 2.**
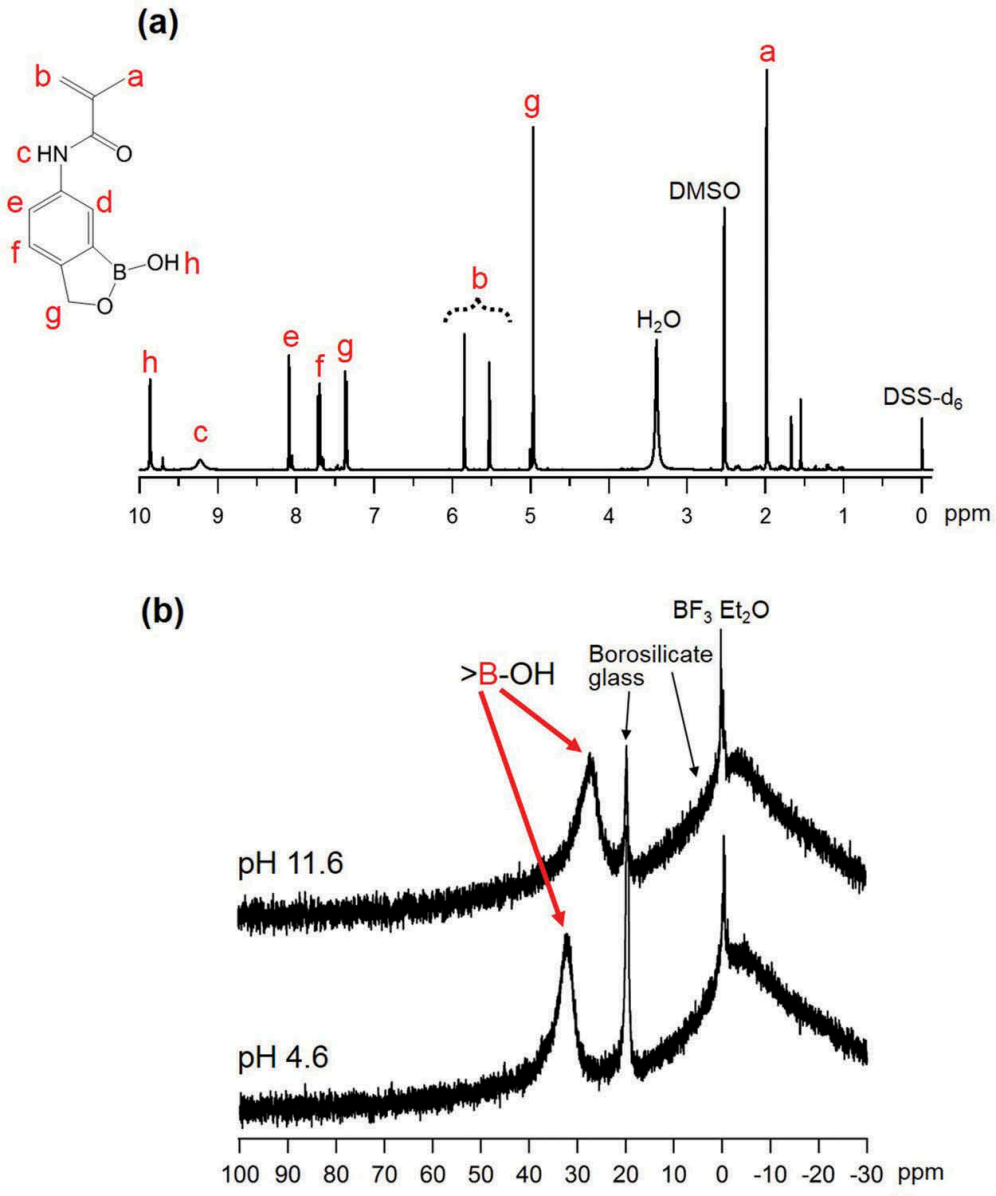
(a) ^1^H-NMR spectrum of MAAmBO monomer in DMSO-d_6_ solvent. The chemical shift of DSS-d_6_ was adopted as the internal reference of 0 ppm. (b) ^11^B-NMR spectra of MAAmBO monomer (5.2 ~ 5.3 mM) in H_2_O/D_2_O (= 9/1) solvent at pH 11.6 and pH 4.6, respectively. The chemical shift of BF_3_·EtO_2_ was adopted as the external reference of 0 ppm.

Figure 2(a) shows the ^1^H-NMR spectrum of the synthesized MAAmBO monomer in DMSO-d_6_ solvent. Since the ^1^H-NMR spectrum was found to be quite similar to previous studies [40,41], the signals at around 2.0 ppm, 5.0 ppm, 5.5 ~ 6.0 ppm, 7.3 ~ 8.0 ppm, 9.2 ppm, and 10.0 ppm were assigned to CH_3_ group in methyl methacrylate (MAAm) (peak; **a**), CH_2_ group in BO (peak; **g**), CH_2_ group in MAAm (peak; **b**), phenyl groups in BO (peaks; **d, e, f**), amide group (peak; **c**), and OH group (peak; **h**), respectively.

We measured ^11^B-NMR spectra of the synthesized MAAmBO in H_2_O/D_2_O (= 9:1) solvent at different pH (4.6 and 11.6) and at an ambient temperature. As shown in Figure 2(b), a broad peak at 27 ppm which was assigned to the boron atom of MAAmBO was observed for the case of pH = 11.6, while the peak is shifted to a lower magnetic field as a result of decrease in pH (pH = 4.6) and appears at 32 ppm. This result suggests that the boronic acid groups in MAAmBO exist as ionized >B(OH)_2_^–^ in a solution with higher pH, but for lower pH, the boronic acid is neutralized as >B-OH. The broad background and sharp peak at around 20 ppm are attributable to the borosilicate glass used as a sample tube and the boric acid produced by hydrolysis of BF_3_, respectively.

**Figure 3.**
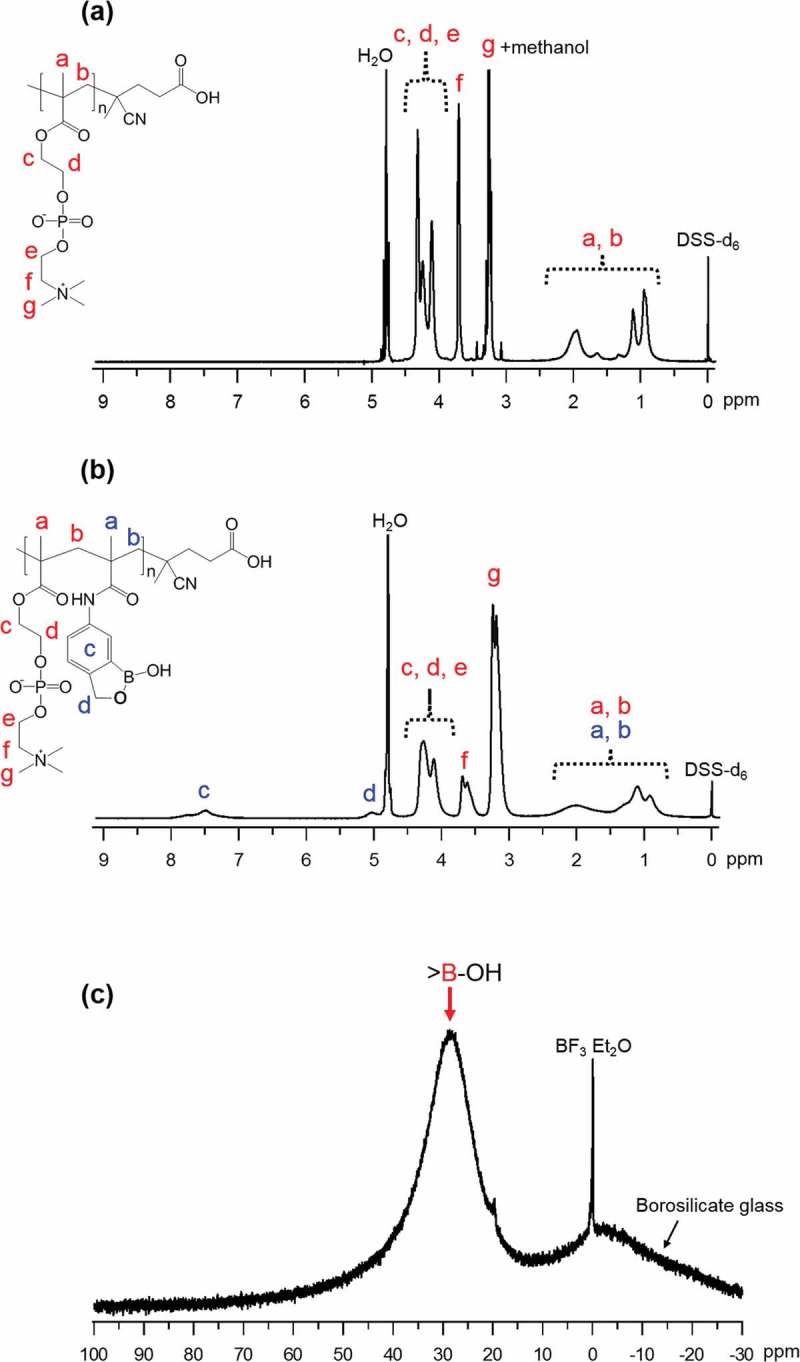
^1^H-NMR spectra of (a) poly[MPC] and (b) poly[MPC-c*o*-MAAmBO] in D_2_O solvent, and (c) ^11^B-NMR spectrum of poly[MPC-c*o*-MAAmBO] in CD_3_OD solvent. DSS-d_6_ and BF_3_·EtO_2_ were used as the internal and the external standards, respectively.

^1^H-NMR spectra of the synthesized poly[MPC] and poly[MPC-*co*-MAAmBO] are shown in Figure 3(a,b), respectively. The ^1^H-NMR peaks related with the poly[MPC] main chain (peaks; **a, b**), oyloxyethyl moiety (peaks; **c, d**), and phosphocholine moiety in MPC (peak; **e, f, g**) are observed at around 1 ~ 2 ppm, 3 ~ 4 ppm, and 4 ~ 4.5 ppm, respectively. On the other hand, in the case of poly[MPC-*co*-MAAmBO], other peaks differing from poly[MPC] are observed at about 5.0 ppm and 7.5 ppm, and they can be assigned to the CH_2_ group in MAAmBO (peak; **D**) and phenyl groups in MAAmBO (peak; **C**), respectively. From the ^1^H-NMR peak area ratio, it was successfully determined that poly[MPC-*co*-MAAmBO] contains 32.4 mol% MAAmBO moiety which is almost comparable with the component of feed solution (30.0 mol%). Moreover, as seen in Figure 3(c), a broad ^11^B-NMR peak of the synthesized poly[MPC-*co*-MAAmBO] is observed in CD_3_OD. The spectrum shape and the chemical shift value of around 27 ppm are similar to those of the MAAmBO monomer. This fact indicates that MAAmBO is not denatured by the polymerization process.

**Figure 4.**
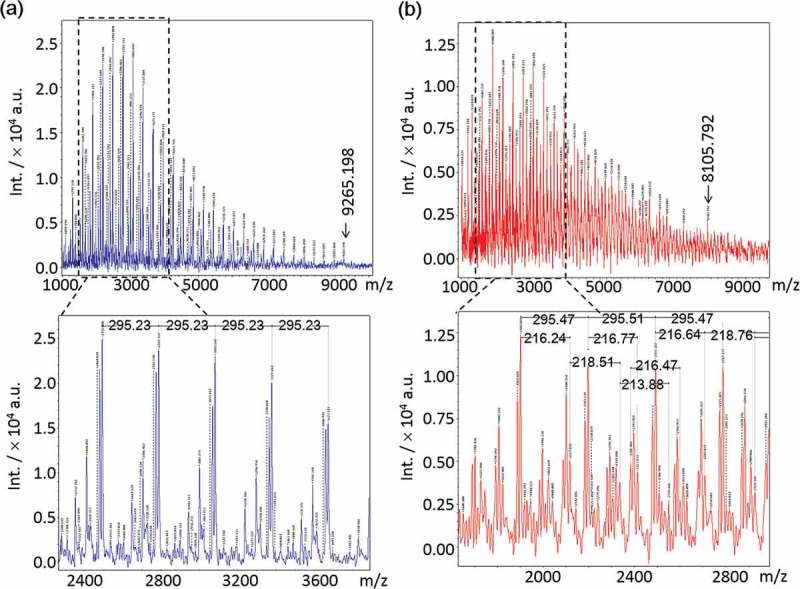
MALDI-TOF mass spectra in reflection mode of (a) poly[MPC] and (b) poly[MPC-c*o*-MAAmBO].

The *M*_n_ values of poly[MPC] and poly[MPC-*co*-MAAmBO] were examined by MALDI-TOF-MS. The repetitive peaks of the poly[MPC] were obtained by each 295 mass unit which is consistent with the MPC monomer. On the other hand, for the case of poly[MPC-*co*-MAAmBO], not only 295 mass units but also 217 mass units corresponding to the MAAmBO monomer were also observed (Figure 4). From the MALDI-TOF mass peak distributions, the *M*_n_ values for poly[MPC] and poly[MPC-*co*-MAAmBO] were roughly estimated to be about 3000 and about 2500, respectively. In addition, the maximum molecular weights for poly[MPC] and poly[MPC-*co*-MAAmBO] were approximately 9300 and 8100, respectively.

### 3.2. Characterization of polymer-modified HVJ-E

Table 2 shows the particle sizes of HVJ-E, HVJ-E/p[MPC], and HVJ-E/p[MPC-MAAmBO] measured by DLS. The averaged diameter of all of the polymer-coated HVJ-E was estimated as 110–150 nm. It seems that the particle size of the polymer-coated HVJ-E is smaller than that of natural HVJ-E (160 nm). Such size reduction of HVJ-E itself by polymer modification demonstrates an opposite tendency as compared to the previously reported polymer CG-HVJ-E-BSH [15]. These results indicate that the spherically structures of HVJ-E are maintained even after the polymer coating, while the effective particle sizes were decreased due to the hyper-hydrophilic biocompatible straight structure of HVJ-E/p[MPC] and HVJ-E/p[MPC-MAAmBO] in aqueous solutions.

**Table 2.** Particle sizes of HVJ-E, HVJ-E/p[MPC], and HVJ-E/p[MPC-*co*-MAAmBO] at each polymer concentration determined by DLS. The sizes are given as mean plus/minus standard deviations. Each error means standard deviation.

Samples	Particle size [nm]
HVJ-E	164 ± 43
HVJ-E/p[MPC-MAAmBO] 100 mg mL^−1^	138 ± 39
HVJ-E/p[MPC-MAAmBO] 50 mg mL^−1^	150 ± 41
HVJ-E/p[MPC-MAAmBO] 10 mg mL^−1^	131 ± 38
HVJ-E/p[MPC] 100 mg mL^−1^	139 ± 40
HVJ-E/p[MPC] 50 mg mL^−1^	116 ± 35
HVJ-E/p[MPC] 10 mg mL^−1^	155 ± 41

**Figure 5.**
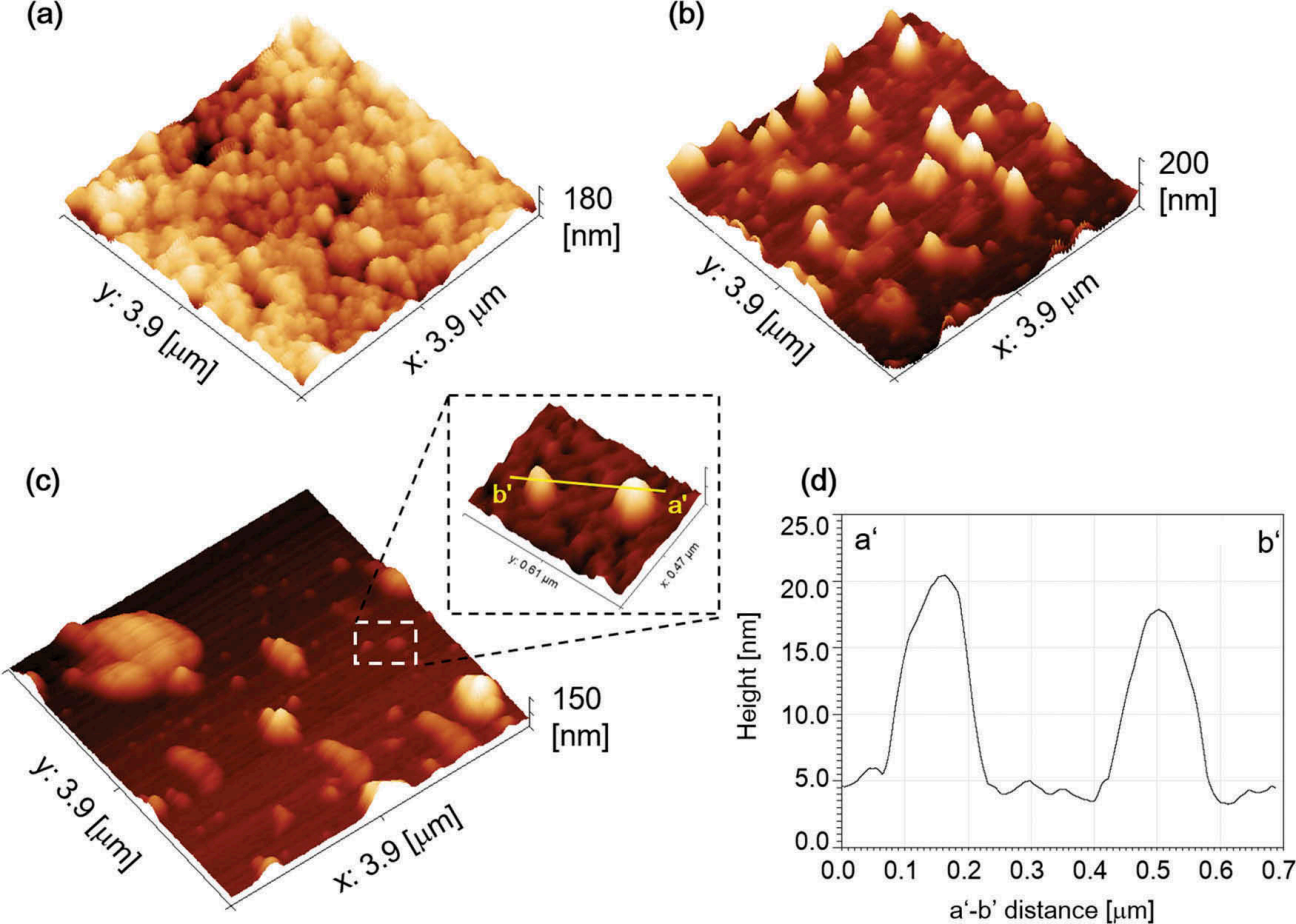
AFM images showing 3D profiles of (a) HVJ-E, (b) HVJ-E/p[MPC], and (c) HVJ-E/p[MPC-MAAmBO] on ODS-modified surfaces, respectively, and (d) a line profile from a’ to b’ in (c) of HVJ-E/p[MPC-MAAmBO].

The shapes of HVJ-E, HVJ-E/p[MPC], and HVJ-E/p[MPC-MAAmBO] nanoparticles were observed by AFM. Since the particle surfaces are hydrophilicity, the solutions containing the particles were dropped on hydrophobic glass surfaces modified by octadecyltrichlorosilane (ODS) vapor and slowly dried for suppressing corruption of the particles. Figure 5(a-c) show the AFM images of HVJ-E, HVJ-E/p[MPC], and HVJ-E/p[MPC-MAAmBO] on the ODS-modified substrates, and the line profile of the HVJ-E/p[MPC-MAAmBO] surface is shown in Figure 5(d). We found that the averaged sizes of HVJ-E and HVJ-E/p[MPC] were about 150 nm which was quite consistent with DLS results. On the other hand, as seen in Figure 5(c–d), in the case of HVJ-E/p[MPC-MAAmBO], the aggregation and the structural deformation were generated under dry conditions because of the hyperhydrophilic structure, resulting in the averaged size of about 180 nm wide and 20 nm height. These results indicate that all of the HVJ-E species well maintained the spherical shapes with 100 nm sizes regardless of polymer modifications.

**Figure 6.**
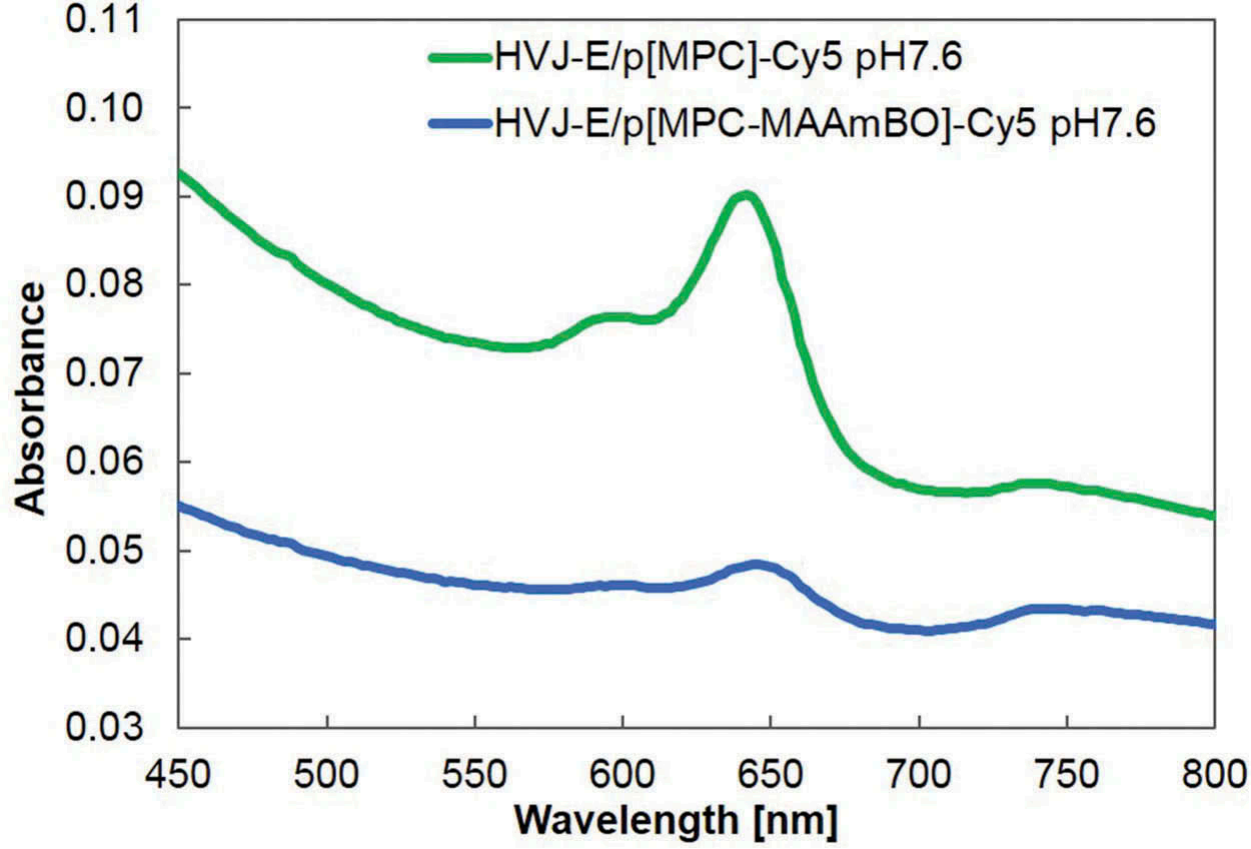
UV-Vis spectra of HVJ-E/Cy5-p[MPC] (green) and HVJ-E/Cy5-p[MPC-MAAmBO] (blue). The spectra were averaged four times.

Figure 6 shows UV-Vis absorption spectra of the supernatant solutions of HVJ-E/Cy5-p[MPC] and HVJ-E/Cy5-p[MPC-MAAmBO] at pH 7.6. The absorption maxima at the wavelengths of 598 and 642 nm, which were assigned to Cy5, were observed for both Cy5-modified HVJ-E polymers. Accordingly, we concluded that HVJ-E may be adequately modified by poly[MPC] and poly[MPC-*co*-MAAmBO] polymers at the concentration range of 100 mg mL^–1^.

**Figure 7.**
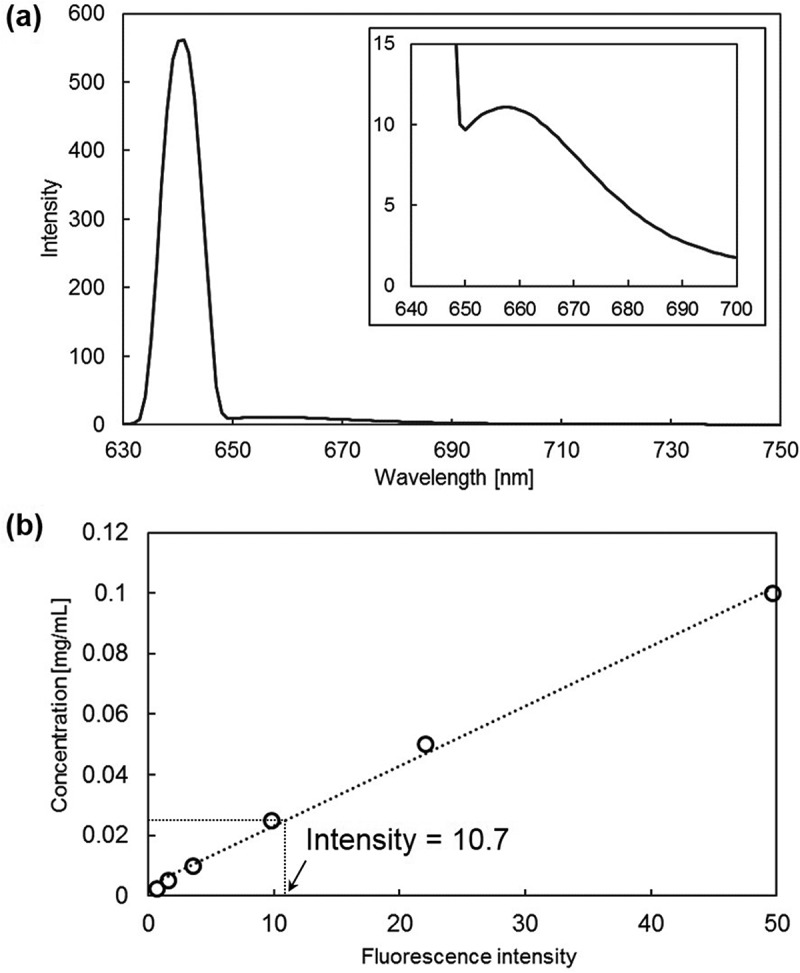
**(a)** Fluorescence spectrum of HVJ-E/Cy5-p[MPC-MAAmBO] from 630 to 750 nm excited by 642 nm. The inset shows a peak assigned to Cy5 at 657 nm. The spectrum was averaged 4 times. **(b)** Calibration curve of fluorescence intensity against concentration of Cy5-p[MPC-*co*-MAAmBO].

Figure 7(a) shows fluorescence spectrum of HVJ-E/Cy5-p[MPC-MAAmBO]. We observed not only a peak corresponding to excitation wavelength (642 nm) but also a peak assigned to Cy5 (657 nm, see the inset in Figure 7(a)), and the fluorescence intensity obtained at 657 nm was determined as 11 ± 3 (*N* = 4). Calibration curve of emission intensity *vs*. Cy5-poly[MPC-*co*-MAAmBO] concentration is shown in Figure 7(b). The concentration of the HVJ-E/Cy5-p[MPC-MAAmBO] could be calculated as 2.44 × 10^–5^ g mL^−1^ by using the calibration curve. From the calculated concentration, the amount of the poly[MPC-*co*-MAAmBO] per single HVJ-E particle could be determined as 1.36 × 10^–13 ~ –14^ g particles^–1^. As already mentioned in the MALDI-TOF and the ^1^H-NMR measurements, the poly[MPC-*co*-MAAmBO] (MPC = 295.3 g mol^–1^, MAAmBO = 216.8 g mol^–1^) has the number-average molecular weight (*M*_n_) of about 2500, and contains 32.4 mol% MAAmBO moiety. Therefore, the number of boron atoms could be estimated as 3.0 per a single poly[MPC-*co*-MAAmBO] copolymer molecule, resulting in 7.22 × 10^20^ boron atoms in one gram of the copolymer. By multiplying the value by 1.36 × 10^–13 ~ –14^ g particles^–1^ of HJV-E, boron atoms of 9.82 × 10^6 ~ 7^ were found to be incorporated on a single HVJ-E particle. This value approaches to previous BSH-encapsulating HVJ-E with 3.72 × 10^10^ boron atoms per particle which was prepared by loading BSH (6667 μg boron) into HVJ-E suspension (1.0 × 10^10^ particles) [15]. Accordingly, the HVJ-E/Cy5-p[MPC-MAAmBO] nanoparticle which enables both high loading of boron atoms and hemolysis suppression is expected to be a promising nanocarrier for BNCT.

UV-Vis absorbance spectrum of D-PBS(-) solution containing the glucose-treated HVJ-E/Cy5-p[MPC-MAAmBO] was measured (N = 5), and compared with non-glucose-treated HVJ-E/Cy5-p[MPC-MAAmBO]. As listed in Table 3, the UV-Vis absorbance at 642 nm of glucose-treated and non-glucose-treated HVJ-E/Cy5-p[MPC-MAAmBO] showed almost the same. From the calculation of the *t* test, the *t*-value and the *p* value were determined as 0.164 and 0.874, respectively. This results verified that there was no statistically significant difference of the UV-Vis absorption between glucose-treated and nonglucose-treated HVJ-E/Cy5-p[MPC-MAAmBO] in D-PBS(-) solutions, and that effect of glucose on the stability of polymer coated on HVJ-E is negligible.

**Table 3.** UV-Vis absorbance of HVJ-E/Cy5-p[MPC-*co*-MAAmBO] at 642 nm by treating D-PBS(-) with D-glucose (2 mg mL^−1^) or without D-glucose. The *p* value was calculated by *t* test, and determined as 0.874 > 0.05. Each error means standard error (*N* = 5).

Samples	UV-Vis absorbance at 642 nm
HVJ-E/p[MPC-MAAmBO] treated by D-PBS(-) with glucose	0.055 + 0.001
HVJ-E/p[MPC-MAAmBO] treated by D-PBS(-) without glucose	0.052 + 0.001

### 3.3. Evaluation of cell viability

Cellular viability was evaluated by alamar blue assay. The fluorescence intensity at 585 nm exited by 540 nm (*N* = 5) of the HepG2 cells treated with poly[MPC-*co*-MAAmBO] was measured, and compared with that of control HepG2 cells without poly[MPC-*co*-MAAmBO]. As listed in Table 4, we found that the cellular viability of poly[MPC-*co*-MAAmBO] case was determined as 107% which is consistent with that of control case. When *t* test was carried out for the difference of the cellular viability between with and without poly[MPC-*co*-MAAmBO], a *p* value of about 0.54 was obtained. This fact indicates that administration of 1 mg mL^–1^ poly[MPC-*co*-MAAmBO] generates no apparent cytotoxicity.

**Table 4.** Cellular viability of HepG2 cells incubated with FluoroBrite DMEM/D-PBS(-) (= 9:1) mixed medium with (control) and without poly[MPC-*co*-MAAmBO] (1 mg mL^−1^). Error bar means standard error (*N* = 5). The *p* value was determined as 0.535 > 0.05.

Samples	Cellular viability [%]
poly[MPC-*co*-MAAmBO] 1mg/mL	107 + 9
Control	100 ± 11

### 3.4. Evaluation of cellar uptake of poly[MPC-co-MAAmBO]

**Figure 8.**
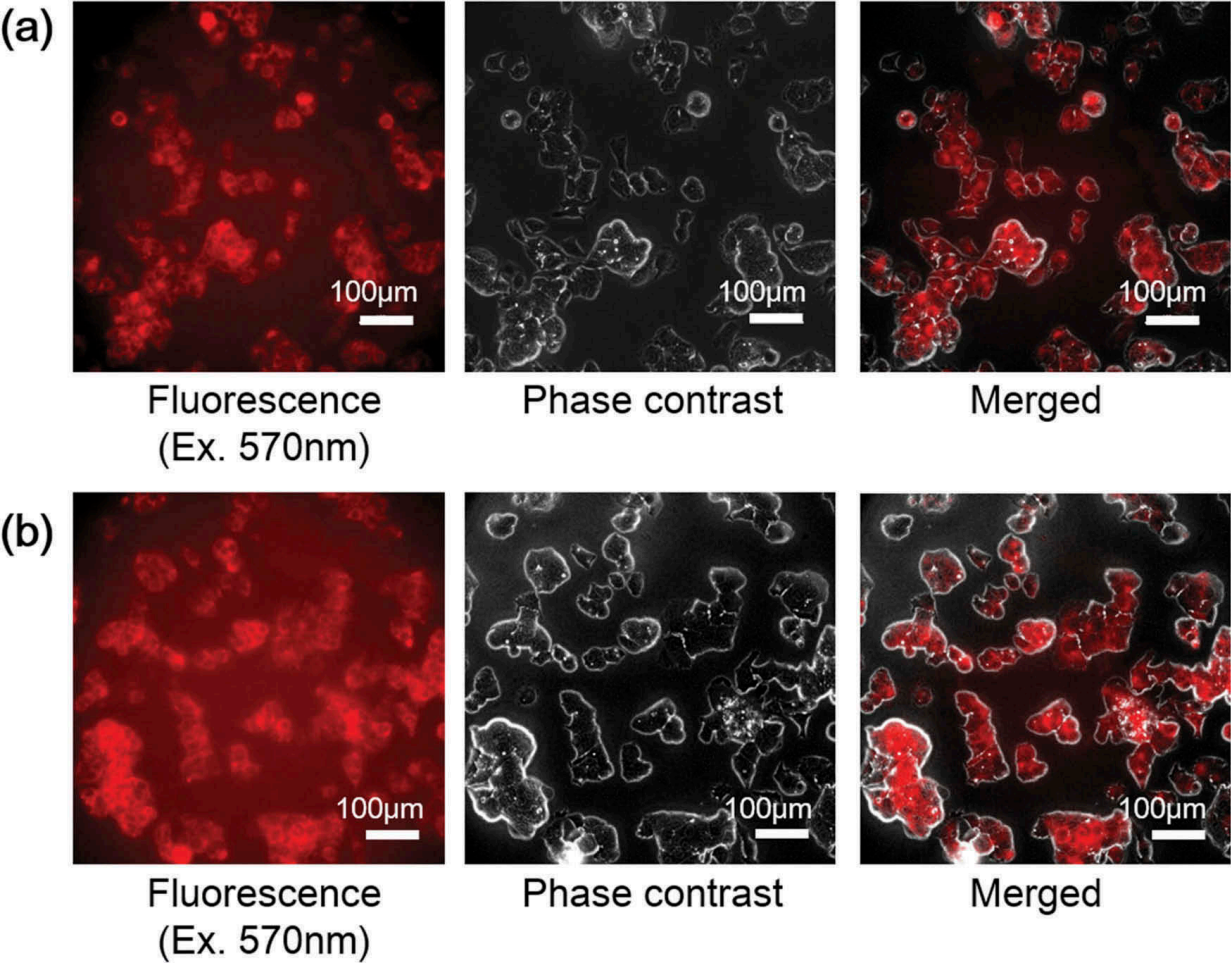
Cellular uptake of Cy5-poly[MPC-*co*-MAAmBO] in HepG2 cells incubated with FluoroBrite DMEM/D-PBS(-) mixed medium (= 49:1) for (a) 45 min and (b) 90 min.

The fluorescence microscopic images of cellular uptake of Cy5-poly[MPC-*co*-MAAmBO] in HepG2 cells for 45 and 90 min are shown in Figure 8(a,b), respectively. The fluorescence color changes showed that significant cytoplasmic localization of the Cy5-poly[MPC-*co*-MAAmBO] was occurred within 45 min, and intracytoplasmic localization of the polymer inside cells was uniformly proceeded during incubation of 90 min. It is expected that such cellular uptake property of poly[MPC-*co*-MAAmBO] play an effective role for enhancing treatment effect of BNCT, since previous PHITS (Particle and Heavy Ion Transport code System) simulation has suggested that the intracellular homogeneous distributions of ^10^B agents is more effective than heterogeneous intra- and intercellular distributions for BNCT treatment [43].

### 3.5. Hemolytic activities of polymer coating

The hemolysis ratio was calculated from the changes in absorbance according to the following equation, where ‘HVJ-E’ and ‘blank’ are obtained from the absorbance of only HJV-E and buffer solution (pH 7.6), respectively.

$$Hemolysis\;ratio\;\left[\%\right]=\text{\textbackslash{}break}\;\frac{Absorbance\;\;of\;sample-Absorbance\;\;of{\;}^{\prime \prime }blank^{\prime \prime }}{Absorbance\;\;of^{\prime \prime }HVJ-E^{\prime \prime }-Absorbance\;\;of^{\prime \prime }blank^{\prime \prime }}\times 100$$

**Figure 9.**
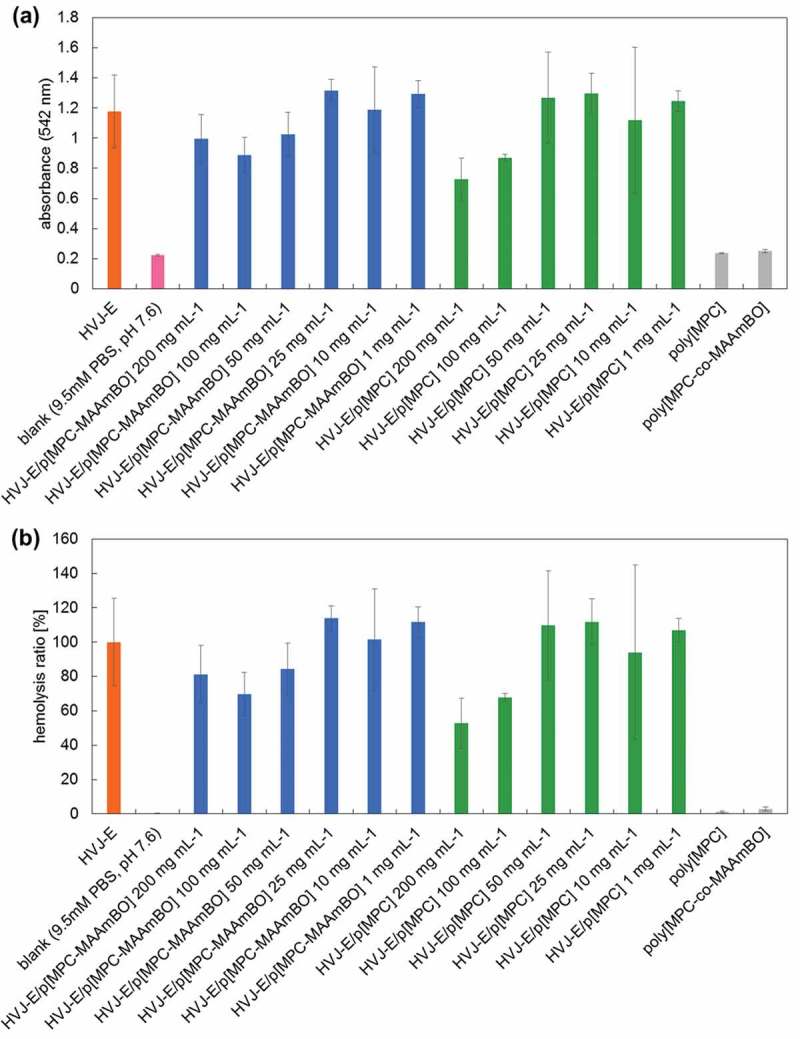
(a) Absorbance at 542 nm of supernatant solutions of 8% chicken erythrocyte suspension after hemolysis test. (b) Calculated hemolysis ratio of each supernatant solution (*N* = 3).

Figure 9(a,b) show the absorbance at 542 nm and the calculated hemolysis ratio of each supernatant solution sample, respectively. We confirmed that the hemolysis ratio was not influenced by poly[MPC] and poly[MPC-*co*-MAAmBO] solution; the ratio was ca. 0.43% and 1.1%, respectively, even in the case of 200 mg mL^−1^. For both HVJ-E/p[MPC] and HVJ-E/p[MPC-MAAmBO], the hemolysis ratio was about 100% at lower concentrations (1 ~ 25 mg mL^–1^) and no hemolysis suppression occurred, but the hemolysis was found to be suppressed in viscous solutions with higher concentrations (100 ~ 200 mg mL^–1^). The highly concentrated polymer solutions were so viscous that the physical adsorption of the polymers onto HVJ-E was promoted. On the other hand, the polymers at lower concentrations (< 25 mg mL^–1^) could not coat the HVJ-E surface and to suppress the hemolysis. Noteworthy, clear differences in the hemolysis ratio between HVJ-E/p[MPC] and HVJ-E/p[MPC-MAAmBO] appeared at the middle concentration (50 mg mL^–1^). The hemolysis ratio radically decreased from 100% to 80% for only HVJ-E/p[MPC-MAAmBO] at 50 mg mL^–1^, but no reduction occurred for HVJ-E/p[MPC]. The reduction of hemolysis by 20% is almost comparable with the case of the LbL method [16]. The results suggest that the optimal concentration (50 mg mL^–1^) of HVJ-E/p[MPC-MAAmBO] makes it possible to modify adequately the HVJ-E surfaces based on the chemical bonding between the benzoxaborole moiety on poly[MPC-*co*-MAAmBO] and the sugar moiety on HVJ-E.

## 4. Conclusions

Benzoboroxole-containing biocompatible polymer, poly[MPC-*co*-MAAmBO], was successfully synthesized, and the molecular structures were characterized by ^1^H- and ^11^B-NMR spectroscopy. The results showed that the synthesized poly[MPC-*co*-MAAmBO] contained 32.4%mol MAAmBO which is almost comparable with the component of feed solution (30.0%mol). Quantitative fluorescence analysis of cyanine5 (Cy5) amine dye-functionalized poly[MPC-co-MAAmBO] coated on HVJ-E (HVJ-E/Cy5-p[MPC-*co*-MAAmBO]) was performed, and the amount of boron atoms in the coating poly[MPC-*co*-MAAmBO] per single HVJ-E particle could be successfully determined as 9.82 × 10^6 ~ 7^. By DLS and AFM measurements, the particle sizes of poly[MPC-*co*-MAAmBO]-coated HVJ-E (HVJ-E/p[MPC-MAAmBO]) with different concentrations were determined to be about 130 ~ 150 nm, slightly smaller than natural HVJ-E itself (160 nm). It was found that the spherical structure of HVJ-E is not degraded by polymer modification, while the hyperhydrophilic biocompatible straight structure of the poly[MPC-*co*-MAAmBO] generates a decrease in the effective particle sizes in aqueous solutions. Moreover, glucose tolerance of the poly[MPC-*co*-MAAmBO] on HVJ-E in a simulated blood solution was examined based on UV-Vis absorbance changes, resulting in that the polymer was steadily coated on the surface of the HVJ-E even in the environment of high glucose level.

In order to clarify biological and cytotoxic effects of the poly[MPC-*co*-MAAmBO], *in vitro* cellular viability and cellular uptake experiments were performed. The differences of fluorescence intensities of HepG2 cells treated with and without poly[MPC-*co*-MAAmBO] obtained by Alamar blue assay exhibited that the polymer has quite low cytotoxicity. The cellular uptake of Cy5-poly[MPC-*co*-MAAmBO] in HepG2 was examined by means of fluorescence microscopy. From the fluorescence color changes, intracytoplasmic localization of the Cy5-poly[MPC-*co*-MAAmBO] into the cells was found to proceed for 45 ~ 90 min.

Hemolysis suppression by coating poly[MPC-*co*-MAAmBO] onto HVJ-E was compared with poly[MPC] by using UV-Vis absorption spectra. We observed that both HVJ-E/p[MPC] and HVJ-E/p[MPC-MAAmBO] at the higher concentrations above 100 mg mL^−1^ induced hemolysis suppression, because of the physisorption effect between the polymer and HVJ-E surfaces. On the other hand, when the polymer concentrations are lower than 25 mg mL^–1^, no hemolysis suppression appears for either HVJ-E/p[MPC] or HVJ-E/p[MPC-MAAmBO]. This phenomenon is due to the polymer concentrations not being enough to coat the HVJ-E surface. However, at the polymer concentration of 50 mg mL^−1^, the hemolysis for only HVJ-E/p[MPC-MAAmBO] was inhibited by 20% as compared with the cases of natural HVJ-E and HVJ-E/p[MPC].

Based on the results, we concluded that biocompatible and low-cytotoxic HVJ-E/p[MPC-MAAmBO], which enables high loading of boron atoms and hemolysis suppression, can be firstly developed. This HVJ-E/p[MPC-MAAmBO] should play an important role in the creation of new boron nanocarriers for BNCT.
